# Sleep & Dreams Group in a Specialist Eating Disorders Unit, an Evaluation

**DOI:** 10.1192/bjo.2022.377

**Published:** 2022-06-20

**Authors:** Fraser Arends, Miles Rush, James FitzGerald

**Affiliations:** 1 Cambridgeshire and Peterborough NHS Foundation Trust, Cambridge, United Kingdom; 2 University of Cambridge, Cambridge, United Kingdom

## Abstract

**Aims:**

Development of an eating disorder in childhood has been shown to predict sleep disturbance in adulthood. Both the National Institute of Health and Care Excellence (NICE) and the wider scientific literature support interventions to help support patients with their sleep. The aim of this project was to evaluate the perceived benefits of the Sleep and Dreams Group to adult patients with anorexia nervosa (AN) on a specialist eating disorders unit.

**Methods:**

Adult patients with severe AN on an inpatient specialist eating disorders unit attended a 6 session, once weekly group on a voluntary basis. The therapeutic group included psychoeducation around sleep hygiene, and an experiential component focusing on sleep/dreaming context of inpatient treatment of severe AN.

**Results:**

All participants(n = 6) either agreed or strongly agreed that their understanding of sleep and dreams had improved. Quality of sleep strongly improved in 20% of participants, however, the remainder reported no significant change in this domain. Despite this, 80% of participants agreed or strongly agreed they got what they wanted from the group, finding the content of the psychoeducation material slightly positive or very positive. The total program length was thought to be appropriate, with 80% describing this as very positive.

**Conclusion:**

The impact of the group on quality of sleep was variable, these results indicate that the value of the group to participants was found in the intergroup processes as evidenced by positive evaluation. This is of particular relevance to severe AN, where interpersonal deficits are often seen and from a treatment perspective in addressing the isolating nature of the disorder. Suggestions for improvement included bolstering the interactive component, and assessing participants regarding eligibility for dream discussion to aid formulation work of the unit.

